# The efficacy of an extended scope physiotherapy clinic in paediatric orthopaedics

**DOI:** 10.1007/s11832-016-0725-9

**Published:** 2016-04-02

**Authors:** Marie O Mir, Ciara Cooney, Cliona O’Sullivan, Catherine Blake, Paula Kelly, Pat Kiely, Jacques Noel, David Moore

**Affiliations:** Physiotherapy Department, Our Lady’s Children’s Hospital, Crumlin, Dublin 12, Ireland; UCD School of Public Health, Physiotherapy and Sport Science, University College Dublin (UCD), Dublin, Ireland; Department of Orthopaedic Surgery, Our Lady’s Children’s Hospital, Crumlin, Dublin, Ireland

**Keywords:** Paediatric orthopaedics, Extended scope physiotherapy, Advanced practice, Triage, Normal variants

## Abstract

**Background:**

The demand for paediatric orthopaedic care is growing, and providing the service required is an increasingly challenging task. Physiotherapist-led triage clinics are utilised in adult orthopaedics to enable the provision of care to patients who may not require a surgical consult. The Physiotherapy Orthopaedic Triage Clinic (POTC) was established in Our Lady’s Children’s Hospital Crumlin in response to increasing demands on the paediatric orthopaedic service. The clinic is run by physiotherapists working in an advanced practice role (APP), and is the first paediatric clinic of its type and scale in the Republic of Ireland.

**Purpose:**

To evaluate the efficacy of the service over the 3-year period from January 2011 to December 2013.

**Methods:**

A review of the prospectively gathered database was performed in order to establish the demographic profile of patients, investigate clinic outcomes, and evaluate the reduction in patient waiting times.

**Results:**

2650 patients were managed by the clinic over the 3-year period. A total of 77 % of patients were managed without consultant intervention. Fifty-three percent of patients were diagnosed as having a normal presentation. The mean waiting time reduced from 101.9 weeks pre-2010 to 15.4 weeks in 2013 for those patients managed by the POTC.

**Conclusion:**

Since its inception, the clinic has significantly reduced waiting times for routine elective paediatric orthopaedic patients while managing the majority of patients independent of surgical opinion. This study shows that the APP can deliver high-quality care in the paediatric orthopaedic setting, benefitting both patients and service.

## Introduction

Pediatric orthopedics is a well-recognised speciality, but it is acknowledged worldwide that it is under-resourced and understaffed [1–3], with a resultant increase in elective waiting lists. A large proportion of those seeking assessment are “the worried well”: children who present for assessment due to parental concern regarding the lower limb, foot and/or gait. These conditions are called “normal or physiological variants” and resolve spontaneously with growth and development. Specialist intervention and overinvestigation should be avoided [4, 5].

The advanced practice physiotherapist (APP) or extended scope physiotherapist (ESP) has been defined as “a clinical specialist, who has the opportunity to develop and demonstrate expertise beyond the currently recognised scope of practice, including some aspect of job enhancement or expansion, involving the areas of extended therapeutics, diagnostics and practice consultation” [6]. The terms have previously been described as interchangeable [7], and APP has been adopted by the Irish Society of Chartered Physiotherapists. APPs are specialist physiotherapists who perform some tasks traditionally performed by doctors, such as patient assessment, diagnosis and treatment, joint injection, removal of plaster of Paris and *K*-wires, and listing for surgery [8]. Drivers for the development of the role have been identified as expanding outpatient waiting lists with concurrent increases in waiting times, large volumes of inappropriate consultant referrals and increasing prevalence of chronic musculoskeletal problems. Several systematic reviews have evaluated the APP role [6, 8–15]. While it is acknowledged that the methodological quality of those studies is poor, all authors have concluded that the majority of evidence supports the role.

The Physiotherapy Orthopaedic Triage Clinic (POTC) was developed in Our Lady’s Children’s Hospital, Crumlin, Dublin (OLCHC) in January 2011 with the premise of providing high-quality care for routine elective referrals that do not require a surgical consult. The hospital is the largest specialist paediatric orthopaedic centre in Ireland, and is both a trauma and tertiary referral centre.

The POTC is staffed by two part-time clinical specialist physiotherapists (one whole-time equivalent), working in an advanced practice role. Four to six clinics are run weekly, concurrent and co-located with consultant orthopaedic surgeons’ clinics. A month’s lead-in time for training and protocol development was allowed, which consisted of both shadowing the consultants in their elective clinics and condition-specific discussions. Assessment proformas were developed, including screening for red and yellow flags utilising a specific paediatric yellow flag tool which had been developed previously in our centre [16].

Ongoing training occurs both during the clinic and with APPs’ attending in-house orthopaedic registrar training. Theoretical training occurs through attendance at specific training days and case discussion with consultants. Links were established with similar clinics in the United Kingdom to ensure the highest standards of service provision. In Ireland, physiotherapists are not licensed prescribers of radiological investigations, so all necessary investigations are ordered by and reviewed with the orthopaedic team. All requests for investigation are initiated by the APP. In all other respects, the APP assesses, diagnoses and formulates management plans autonomously, and any request for consultant opinion is initiated at the APP’s discretion.

Elective referrals to the hospital are paper-based, and referrals are triaged to the POTC by the consultants. Initial inclusion criteria were restricted to normal variants and were expanded as the clinic developed to include idiopathic toe walkers, curly toes, orthopaedic gait concerns and lower limb pain of mechanical origin. Exclusion criteria are as follows: low back pain, referrals from other hospital consultants, lower limb conditions with specific diagnoses such as Perthes disease, requests for surgical opinion and referrals suggestive of neuromuscular disorders, infective, inflammatory or malignant disease.

The aims of this study were to evaluate the reduction in waiting times for the elective patient cohort managed in the POTC, and investigate the clinic outcomes, particularly the proportion of patients who were managed without consultant referral.

## Materials and methods

This was a prospective longitudinal cohort study conducted over a 3-year period from January 2011 to December 2013. Prospective waiting times between date of referral and date of initial assessment were calculated. The resulting sample included children referred from as far back as 2007 through to 2013.

### Data collection

An electronic database was established to prospectively record the following variables: date of birth, gender, date of referral, date of first appointment offered, referring diagnosis, diagnosis after assessment in the POTC and clinical care pathway outcomes. To allow for statistical analyses, referring diagnosis was categorised as one of the following: pes planus, rotational variation, limb deformity, toe deformity, pain, gait abnormality and other. Similarly, diagnosis after assessment in the POTC was recorded as one of ten categorical variables, which were established in accordance with ICD-10 diagnostic codes. Four clinical care pathway outcomes were established.

Data cleaning was performed and patient charts were reviewed to obtain missing data. All data recorded were cross-referenced with the hospital patient administration system (PAS) to ensure the accuracy of the data.

### Statistical analyses

Descriptive statistics were calculated using SPSS 20.0. To facilitate further analysis, a cross-tabulation was performed to explore the relationship between (1) age and diagnosis and (2) diagnosis and clinical pathway outcome.

Waiting-time reduction was computed by ascertaining the difference in time (weeks) between date of referral and date of initial appointment offered for each patient. The minimum, maximum and mean waiting time were calculated for each referral year. One-way ANOVA was performed to establish if there was a statistically significant difference in mean waiting times year on year. Pairwise comparisons of mean waiting times for the yearly intervals was performed with the post hoc Tukey test.

## Results

A total of 2650 patients were offered first appointments with the POTC between January 2011 and December 2013. Six hundred and sixty-two (23 %) patients failed to attend their appointment. The remaining 2028 subjects form the basis of this study.

### Demographics and reason for referral

Of those who presented, 53 % (*n* = 1406) were male, and the age range was from 0.4 to 19 years with a mean age of 6.4 years (SD 4.5 years). Fifty-five percent of patients were aged 5 years and under. The most frequent cause of referral was rotational variation (Table 1). Conditions within the spectrum of “normal or physiological variants” constituted 44 % of referrals.

**Table 1:** Demographic details and referral diagnosis of patients

Demographics	*N*	(%)
Gender		
Male	1406	(53)
Female	1244	(47)
Age (years)		
Mean (±SD)	6.4 (±4.5)	
Referral diagnosis		
Rotational variation	665	(25)
Pain	613	(23)
Pes planus	340	(13)
Gait abnormality	305	(12
Toe deformity	226	(8)
Limb deformity	164	(6)
Other	337	(13)

### POTC diagnosis

**Fig. 1:**
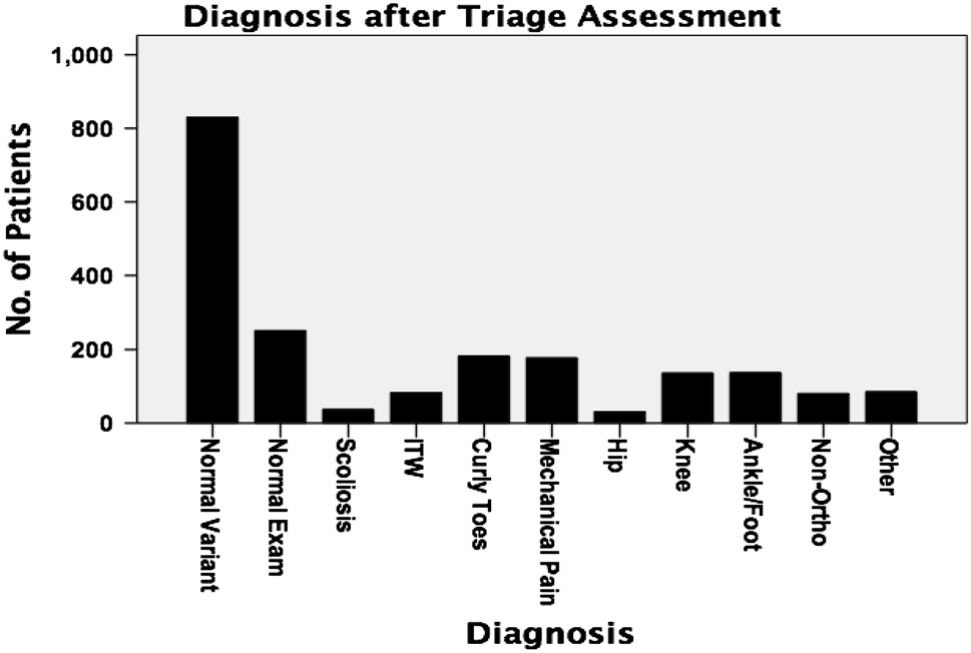
Distribution of patients based on diagnosis after assessment in the Physiotherapy Orthopaedic Triage Clinic (POTC) (*N* = 2028). *ITW* idiopathic toe walkers

Figure 1 presents the diagnostic categories of patients post assessment and the frequency per category. Normal presentation was the most common diagnosis (53 %). Non-orthopaedic patients (e.g. suspected developmental delay, developmental coordination disorder, neurological or inflammatory disorders) accounted for 3 % of patients, and were referred directly to appropriate specialities after discussion with the orthopaedic consultant.

**Fig. 2:**
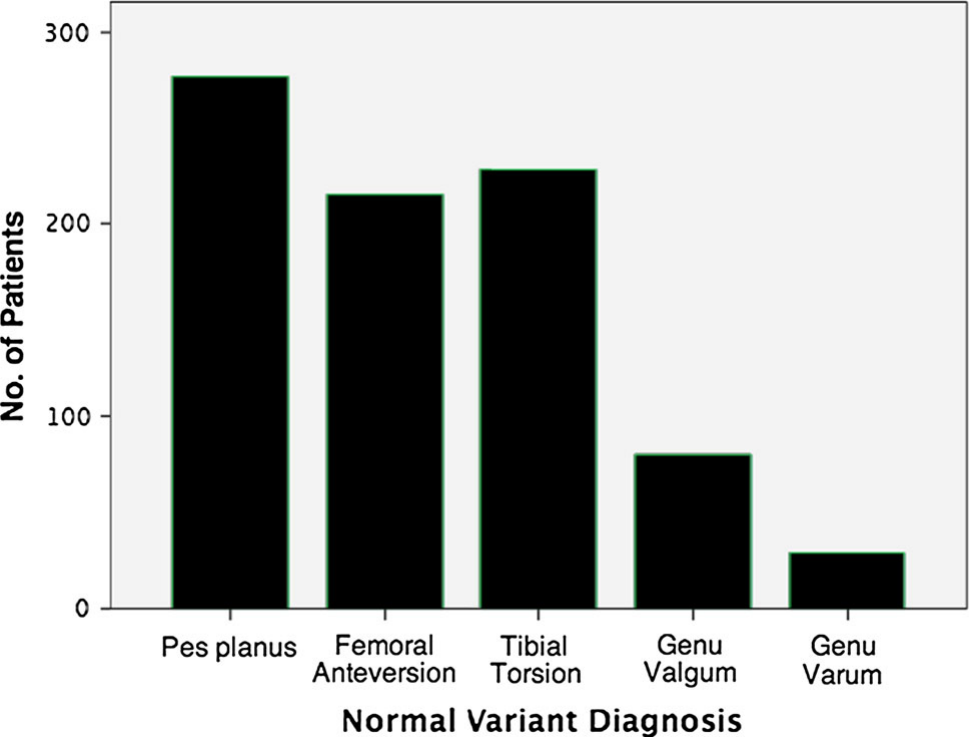
Distribution of most common diagnoses within the normal-presentation population, based on assessment at POTC (*N* = 830)

Within the normal variant population, the most common diagnosis was pes planus and least common was genu varum (Fig. 2).

Cross-tabulations were performed between age (at time of assessment) and diagnosis (Table 2). Normal presentation was most common under 5 years of age (75 %). Scoliosis patients typically presented at ≥12 years (57 %). Fifty percent of idiopathic toe walkers and curly toes presented at 3–5 years old. Mechanical pain was most typically seen in those ≥6 years (72 %). While knee disorders (84 %) and ankle/foot disorders (71 %) were most commonly seen in those >6 years of age, there was an even spread in hip pathology across all age groups. A similarly even spread was also demonstrated in “other” conditions, with the highest rate of presentation for non-orthopaedic conditions (43 %) occurring in the 3–5 years age group.

**Table 2:** Diagnosis after triage assessment cross-tabulated against age at assessment (*n* = 2028, representing total number of patients who presented for initial assessment)

Diagnosis (Dx)	Age grouped into 4 categories				Total
	12+ years	6–11 years	3–5 years	0–2 years	
	*n* (%)	*n* (%)	*n* (%)	*n* (%)	*n*
Normal	83 (8)	183 (17)	488 (45)	326 (30)	1080
Scoliosis	21 (57)	8 (22)	5 (13)	3 (8)	37
ITW^♦^	2 (2)	24 (29)	42 (51)	15 (18)	83
Curly toes	21 (11)	34 (19)	91 (50)	36 (20)	182
Mechanical pain	80 (45)	48 (27)	45 (26)	4 (2)	177
Hip pathology	11 (35)	7 (23)	8 (26)	5 (16)	31
Knee pathology	84 (62)	30 (22)	11 (8)	11 (8)	136
Ankle/foot pathology	44 (32)	53 (39)	16 (12)	24 (17)	137
Non-ortho. condition	12 (15)	19 (24)	34 (42)	15 (19)	80
Other	19 (22)	19 (22)	29 (35)	18 (21)	85

^♦^ *ITW* idiopathic toe walker

### Patient clinical care pathway outcomes

Clinical care pathway outcomes are reported in Table 3. A total of 77 % of patients were managed independently without consultant intervention. The most frequent outcome was discharge back to GP/physiotherapy after initial assessment (69 %). Eight percent of patients were advised to return for review at the POTC.

**Table 3:** Clinical outcomes following initial assessment at POTC

Clinical outcome	Total patients	NV^a^ population
	*n* (%)	*n* (%)
D/C back to referrer/physiotherapy	1399 (69)	1011 (94)
Review at POTC^¶^	159 (8)	35 (3)
Refer to orthopaedic clinic	425 (21)	33 (3)
Refer to other speciality	45 (2)	0 (0)

^¶^ *POTC* Physiotherapy Orthopaedic Triage Clinic

^a^Indicates normal variant population only, based on the diagnosis being the same after assessment at POTC

Physiotherapy referrals were made for 387 patients, of whom 159 returned for review in either the POTC (*n* = 95) or the consultant clinic (*n* = 64). Direct referrals to orthopaedics comprised 18 % of the patients seen, with a further 3 % attending orthopaedics after a course of physiotherapy.

In total, 45 patients were referred directly from POTC to another specialty such as paediatrics, neurology and rheumatology.

Examining the normal variant population specifically, the independent management rate is much higher (Table 3), with only 3 % of this cohort requiring consultant review. The remainder were managed independently, and 94 % of the total were discharged after their initial assessment.

The results of a cross-tabulation between diagnosis and outcome after assessment can be seen in Table 4. Curly toes had an even division of management, with 50 % being discharged and the remainder referred for consultant opinion. This is in line with international results [17, 18]. Diagnoses where >50 % of the patients were referred for consultant opinion were scoliosis (67 %), hip pathology (90 %) and “other” disorders (67 %). Mechanical pain was typically referred to physiotherapy (60 %), as were ankle/foot (47 %) and knee disorders (60 %). Non-orthopaedic conditions were commonly referred for review, either with the orthopaedic team (35 %) or directly to other services (41 %).

**Table 4:** Cross-tabulation of diagnosis after triage assessment versus outcome (4 categories)

Diagnosis	Outcome				Total^a^
	D/C to GP/PT^¤^	R/V at POTC	Ortho R/V	Refer to other service	
	*n* (%)^b^	*n* (%)	*n* (%)	*n* (%)	
Normal	1011 (94)	35 (3)	33 (3)	0	1080
Scoliosis	6 (16)	6 (16)	25 (68)	0	37
ITW^♦^	26 (31)	34 (41)	22 (27)	1 (1)	83
Curly toes	91 (50)	34 (1)	87 (48)	0	182
Mechanical pain	103 (58)	36 (20)	37 (21)	1 (1)	177
Hip disorder	2 (7)	1 (3)	28 (90)	0	31
Knee disorder	78 (57)	14 (10)	44 (33)	0	136
Ankle/foot disorder	59 (43)	12 (9)	64 (47)	2 (1)	137
Non-ortho. condition	12 (15)	7 (9)	28 (35)	33 (41)	80
Other	11 (13)	11 (13)	57 (67)	6 (7)	85

^¤^ *PT* physiotherapy, ^♦^ *ITW* idiopathic toe walker

^a^Represents the total number of patients within each diagnostic category

^b^Represents the % of patients within each diagnostic category (in italics)

### Waiting time reduction

**Fig. 3:**
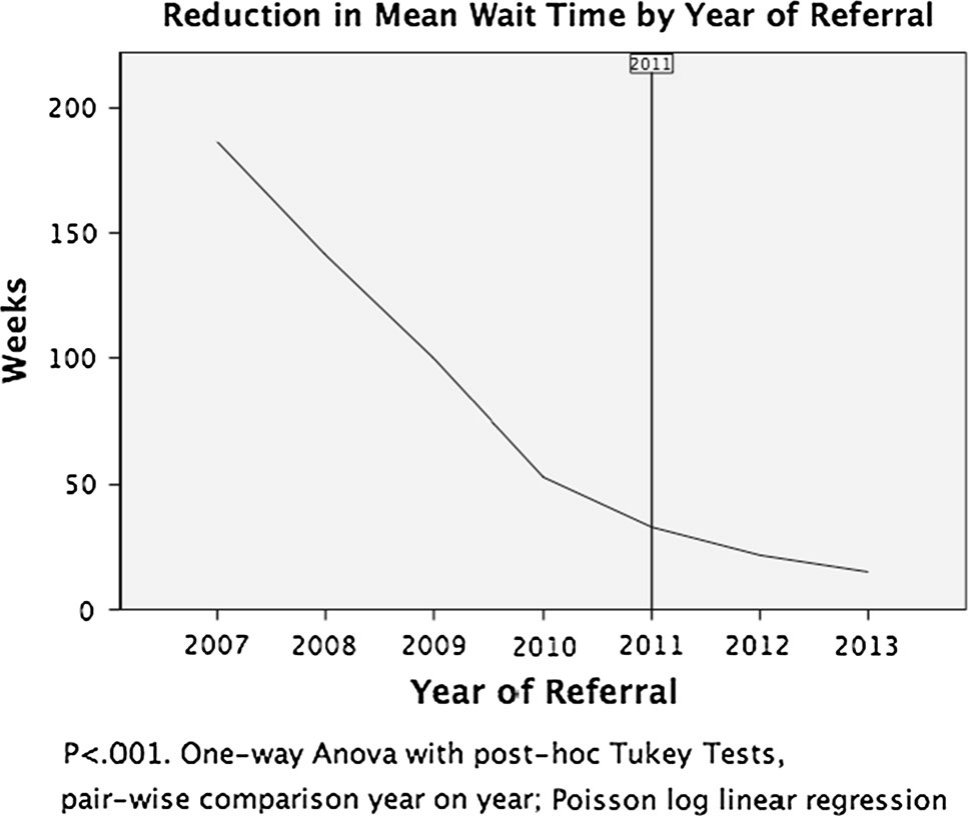
Line graph presenting the reduction in mean waiting time based on year of referral. The *horizontal axis* indicates the commencement of the POTC

Mean waiting time was reduced from 101.9 weeks pre-2010 to 15.4 weeks over the 3-year period for the patient cohort deemed suitable for the POTC (Fig. 3). As the data were widely dispersed, a one-way ANOVA test of the means was calculated, which showed a statistically significant (*p* < 0.001) reduction in mean waiting time. Post-hoc Tukey tests were then performed which demonstrated that the statistically significant reduction held for each pairwise year-on-year comparison, including 2012–2013.

## Discussion

There is a marked absence of literature on paediatric APP. This study is the first to report on APP in the Irish paediatric setting, and is only the second article to report on APP in paediatric orthopaedics, despite the practice being widespread in several countries, with the last complete study published in 2003. Belthur et al. [19] described a service led by an extended scope physiotherapist managing elective non-urgent paediatric orthopaedic referrals. Median waiting time for patients was reduced from 72 to 5 weeks, and 93 % of patients were managed without direct consultant intervention. In an abstract published in 2009 [20], Lipscombe et al. reported similar results in a normal variant clinic managed by an ESP, with 94.7 % of the patients managed independently by the ESP.

The results of this current study are comparable to those obtained previously, and demonstrate that the addition of an APP triage clinic significantly reduced waiting times for this patient cohort while managing the majority of patients autonomously.

Our findings that over 53 % of the patients were “normal presentation” patients concurs with previously published reports that a majority of elective referrals to paediatric orthopaedic specialists are for variants of normal development [33], and most are discharged after initial consultation [1, 2, 6–9, 33].

This study provides us with important demographic information for a large cohort of elective patients in terms of most common diagnoses, the age at which patient presents and typical outcomes for each diagnosis. This is important for developing clinical treatment pathways to allow for appropriate patient management from first presentation to their primary care physician.

Several authors have cited a lack of confidence in musculoskeletal diagnostic skills amongst primary care physicians as a reason for the high rate of unnecessary referrals [21–28], and have called for improved musculoskeletal education as part of entry-level medical education. The American Academy of Paediatrics in 2002 published guidelines for primary care physicians on referral for specialist opinion [29], but subsequent studies found that this publication has had no effect on referral patterns [1, 30]. It is within this context that new strategies are required to provide effective high-quality care for patients. This study demonstrates that APP triage is beneficial to both patients and services.

A stated aim of APP clinics is to provide effective assessment and care to patients who do not require a surgical consult. Within condition/joint-specific clinics, the rate of autonomous management varies greatly. It has been reported that shoulder conditions require consultant review more often (81 %) than either knee (34 %) or back disorders (11 %) [31]. None of the adult literature has reported independent management rates as high as those for normal variant clinics [19, 20]. It follows that the APP role is particularly effective in paediatric orthopaedics, especially in the management of normal variants.

Currently, Ireland is far behind international standards of orthopaedic staffing, with 1 paediatric orthopaedic surgeon per 47,000 per capita, while the ideal is 1 per 17,000 head of population (Moore, 2016, personal communication). While the Irish Health Service Executive (HSE) is moving towards a primary health care system to manage most nonsurgical conditions, the reality is that a large proportion of the population continue to seek tertiary-level referrals. With up to 50 % of elective referrals to paediatric orthopaedics being made up of normal variants [2, 24, 27, 30, 32], these nonsurgical candidates require an alternate system of management that provides efficient, high-quality assessment and management, with ready access to surgical opinion if necessary. The success of APP in adult orthopaedics, and the close working relationships of physiotherapists with orthopaedic surgeons in our centre, identified the APP model as the most appropriate service strategy.

As the paediatric APP role is a new departure in our tertiary-level clinic and within the Irish HSE, a Standard Operating Procedure was developed which identified that clinical governance for the POTC lies with the consultant with whom the clinic was interfacing. The APP is covered by the hospital’s clinical indemnity scheme. It should be noted that physiotherapists have long had direct access rights in the Republic of Ireland and are recognised diagnosticians in our healthcare system.

There are several limitations of this study. Firstly, there is bias in that the primary researcher is one of the clinicians in the clinic and was involved in the collection of all data and establishing inclusion/exclusion criteria. This may have influenced the type of patient deemed suitable for the clinic and the resultant outcome. A further limitation is that it is unknown if some patients failed to present because they had been offered an appointment with a physiotherapist rather than an orthopaedic consultant.

Longitudinal follow-up on patients was not conducted in this study. Further study is underway on patient outcomes, as well as the re-referral rate/referral for second opinion, to evaluate any possible erroneous decisions. However, no patients seen at the POTC subsequently presented with malignancy or infective disorders in our tertiary facility.

While this study showed a significant reduction in mean waiting time during the three years, there is a skew in the results. As the clinic became established, inclusion criteria were broadened, and the waiting list was reviewed to identify those waiting in excess of a year who met the new criteria. Therefore, mean waiting times were negatively impacted by long waiters being offered appointments with the POTC at various points over the three years of the study.

An evaluation of the reduction in time needed for consultant appointments was not conducted in this study; this will be addressed in a follow-up study that also looks at diagnostic agreement rates. Further study is planned to evaluate stakeholder and client satisfaction, and to carry out economic cost–benefit analyses that can provide clearer evidence of the efficacy and value of the APP role in paediatric orthopaedics.

While it is not within the scope of the present study, many systematic reviews on the development of the ESP/APP role have called for the identification of competencies and standardised training [6–8, 10, 13, 14, 33], and this is an identified need in the area of paediatric orthopaedics. Many paediatric physiotherapists are highly specialised in the management of neurologic presentations, but may lack experience with musculoskeletal pathologies. Future study is required to identify the competencies needed for APPs in paediatric orthopaedics to allow for the development of standardised education programmes.

In conclusion, an increasing demand for and consequent strain on our paediatric orthopaedic services was met locally by increasing the provision of new patient appointments (through the POTC), developing condition-specific clinics, and providing graduated access to tertiary specialist surgical opinion. The specialist physiotherapists are in effect a filter between primary and secondary care systems.

This study demonstrates that routine elective paediatric orthopaedic referrals can be successfully managed by an APP without recourse to consultant intervention. Waiting times for this patient cohort were reduced significantly while the capacity to see new elective patients was increased. These results firmly support the extended scope physiotherapist role in paediatric orthopaedics as an adjunct to paediatric orthopaedic services.
